# Current smoking status may be associated with overt albuminuria in female patients with type 1 diabetes mellitus: a cross-sectional study

**DOI:** 10.1186/1617-9625-10-12

**Published:** 2012-08-10

**Authors:** Kenta Okada, Jun-ichi Osuga, Kazuhiko Kotani, Hiroaki Yagyu, Michiaki Miyamoto, Shoichiro Nagasaka, Shun Ishibashi

**Affiliations:** 1 Department of Internal Medicine, Division of Endocrinology and Metabolism, Jichi Medical University, Tochigi 320-0498, Japan; 2 Department of Clinical Laboratory Medicine, Jichi Medical University, Tochigi 320-0498, Japan

**Keywords:** Type 1 diabetes mellitus, Current smoking, Overt albuminuria

## Abstract

**Background:**

There are very few clinical reports that have compared the association between cigarette smoking and microangiopathy in Asian patients with type 1 diabetes mellitus (T1DM). The objective of this study was to assess the relationships between urinary protein concentrations and smoking and gender-based risk factors among patients with T1DM.

**Methods:**

A cross-sectional study of 259 patients with T1DM (men/women = 90/169; mean age, 50.7 years) who visited our hospital for more than 1 year between October 2010 and April 2011 was conducted. Participants completed a questionnaire about their smoking habits. Patient characteristics included gender, age, body mass index, blood pressure, hemoglobin A1c, lipid parameters, and microangiopathy. Diabetic nephropathy (DN) was categorized as normoalbuminuria (NA), microalbuminuria (MA), or overt albuminuria (OA) on the basis of the following urinary albumin/creatinine ratio (ACR) levels: NA, ACR levels less than 30 mg/g creatinine (Cr); MA, ACR levels between 30 and 299 mg/g Cr; and OA, ACR levels over 300 mg/g Cr.

**Results:**

The percentages of current nonsmokers and current smokers with T1DM were 73.0% (n = 189) and 27.0% (n = 70), respectively. In addition, the percentage of males was higher than that of females (52.2% versus 13.6%) in the current smoking population. The percentage of DN was 61.8% (n = 160) in patients with NA, 21.6% (n = 56) in patients with MA, and 16.6% (n = 43) in patients with OA. The percentage of males among OA patients was also higher than that of females (24.4% versus 12.4%). However, current smoking status was associated with OA in females with T1DM only [unadjusted odds ratio (OR), 4.13; 95% confidence interval (CI), 1.45–11.73, *P* < 0.01; multivariate-adjusted OR, 5.41; 95% CI, 1.69–17.30, *P* < 0.01].

**Conclusions:**

Based on our results in this cross-sectional study of Asian patients with T1DM, smoking might be a risk factor for OA among female patients. Further research is needed of these gender-specific results.

## Background

Type 1 diabetes mellitus (T1DM) is common in young people [1-3]. Because high blood glucose levels begin at a young age in T1DM, the early management of risk factors is important for preventing diabetic complications in young patients with T1DM. Overt albuminuria (OA) is a diabetic complication, and diabetic nephropathy (DN) is a possible predictor of renal failure and mortality in patients with T1DM [4,5]. The development of DN has been shown to be associated with the duration of diabetes, smoking, hemoglobin A1c (HbA1c) levels, blood pressure levels, and dyslipidemia in T1DM patients [6,7].

It has recently been reported that sex is an important factor in the prevalence of smoking and cardiovascular disease [8]. At present, only limited information is available regarding the association between DN and the gender-based smoking status of T1DM patients [9]. The EURODIAB IDDM Complications Study reported that a current smoking status is positively associated with the progression of DN in male and female patients with T1DM [9]. Because there have been very limited clinical studies in the Asia-Pacific regions in particular, more studies of the influence of gender on smoking habits in DN patients are required. Therefore, the present study aimed to examine risk factors, including current smoking habits that can influence the gender-based presence of OA in Japanese patients with T1DM.

### Subjects and methods

A total of 259 patients with T1DM (men/women = 90/169; mean age = 50.7 ± 16.8 years; duration of diabetes = 17.0 ± 11.0 years) who visited our hospital for more than 1 year between October 2010 and April 2011 were examined. The eligibility criteria were as follows: age > 18 years, the absence of acute ketosis, hypoglycemia, and pregnancy. T1DM was diagnosed according to the Position Statement of the American Diabetes Association [10]. T1DM was defined with the following criteria: absolute insulin deficiency (urine C-peptide levels < 20 μg/day, fasting serum C-peptide levels < 0.5 μg/mL, or serum C-peptide levels < 1.0 μg/mL after loading), susceptibility to ketosis, and requirement of insulin therapy within 1 year after diagnosis; all patients in this study required insulin injections. Smoking habits were confirmed by self-reports. Current smoking status was considered as either smoking or not smoking. The study protocol was approved by the Jichi Medical University Ethical Committee.

The body mass index (BMI) and systolic/diastolic blood pressure were measured in each patient. Blood and urinary samples were collected at the outpatient clinic in order to measure the levels of the following parameters: HbA1c, total cholesterol (TC), triglyceride (TG), and high-density lipoprotein cholesterol (HDL-C). A high-performance liquid chromatography (HA8131; Kyoto Daiichi Kagaku Co., Ltd., Tokyo, Japan) system was used to measure HbA1c levels according to previous Japanese standard substance and measurement methods. National Glycohemoglobin Standardization Program-equivalent values were obtained by using the following equation: HbA1c = HbA1c (the Japan Diabetes Society) + 0.4 [11]. TC, TG, and HDL-C levels were measured by enzymatic methods with an automatic analyzer (Hitachi Co., Ltd., Tokyo, Japan).

Urinary albumin levels were measured with the turbidimetric immunoassay method using a commercially available kit (Auto Wako Albumin; Wako Pure Chemical Industries, Ltd., Osaka, Japan), and urinary creatinine levels were determined by an enzymatic method in order to calculate the urinary albumin/creatinine ratio (ACR). DN was classified as normoalbuminuria (NA), microalbuminuria (MA), or OA on the basis of the following ACR levels: NA, ACR levels less than 30 mg/g creatinine (Cr); MA, ACR levels between 30 and 299 mg/g Cr; and OA, ACR levels over 300 mg/g Cr [12]. Diabetic retinopathy and neuropathy were either present or absent.

The data are presented as mean ± standard deviation (SD) by sex. Comparisons between the groups were made with unpaired *t*-tests and Chi-square tests. Unadjusted, age-adjusted, and multivariate-adjusted logistic regression analyses were performed in order to estimate the factors associated with urinary albuminuria. The multivariate-adjusted logistic regression analysis models included age, BMI, systolic/diastolic blood pressure, HbA1c levels, TC levels, TG levels, HDL-C levels, and current smoking status at baseline as independent variables. Statistical significance was set at *P* values less than 0.05. All analyses were performed with the SPSS II software package (IBM Corporation, Armonk, NY).

## Results

The clinical characteristics of the current smokers and nonsmokers are shown by gender in Table 1. A total of 73% (n = 189) of the study population were nonsmokers, while 27.0% (n = 70) reported themselves as current smokers. Compared to males, females constituted a significantly lower percentage of current smokers (52.2% versus 13.6%, *P* < 0.01). In the nonsmoking group, females had significantly higher HDL-C levels than males, while a similar but nonsignificant difference in HDL-C levels was seen in current smokers. In addition, the percentage of nonsmoking patients with OA was significantly higher in males than in females. For females, the percentage of patients with OA was significantly higher in current smokers than in nonsmokers.

**Table 1:** Clinical characteristics

**Current smoking**	**Male**		**Female**					
	**-**	**+**	**-**	**+**	***P***_**1**_	***P***_**2**_	***P***_**3**_	***P***_**4**_
Total number (%)	43 (47.8)	47 (52.2)	146 (86.4)	23 (13.6)	-	-	-	-
Age (years)	53.2 ± 16.9	49.4 ± 12.6	51.3 ± 18.3	45.5 ± 12.6	0.54	0.24	0.23	0.15
Body mass index (kg/m^2^)	22.4 ± 2.8	22.7 ± 3.4	22.6 ± 3.9	21.8 ± 4.1	0.76	0.32	0.62	0.36
Systolic blood pressure (mmHg)	127.4 ± 24.0	132.3 ± 21.7	125.7 ± 18.8	123.4 ± 14.6	0.62	0.08	0.31	0.58
Diastolic blood pressure (mmHg)	70.8 ± 11.3	74.5 ± 14.6	71.2 ± 12.4	74.3 ± 10.2	0.83	0.94	0.18	0.26
HbA1c (%)	8.4 ± 2.0	8.3 ± 1.7	8.3 ± 2.0	8.7 ± 2.2	0.66	0.34	0.73	0.30
Total cholesterol (mg/dL)	189.7 ± 32.1	187.5 ± 41.4	199.1 ± 37.5	197.3 ± 35.9	0.14	0.34	0.78	0.84
Triglyceride (mg/dL)	96.9 ± 60.2	116.2 ± 81.9	94.7 ± 91.3	98.0 ± 58.5	0.88	0.34	0.21	0.87
HDL-cholesterol (mg/dL)	68.2 ± 18.1	67.2 ± 22.1	75.9 ± 19.1	74.3 ± 13.2	0.02	0.16	0.82	0.70
Complication, n (%)								
Retinopathy	24 (55.8)	26 (55.3)	64 (43.8)	11 (47.8)	0.17	0.56	0.96	0.72
Neuropathy	28 (65.1)	28 (59.6)	86 (58.9)	15 (65.2)	0.46	0.65	0.59	0.57
Nephropathy								
Normoalbuminuria	25 (58.1)	25 (53.2)	96 (65.8)	14 (60.9)	0.36	0.54	0.64	0.65
Microalbuminuria	9 (20.9)	9 (19.1)	36 (24.7)	2 (8.7)	0.61	0.26	0.83	0.09
Overt albuminuria	9 (20.9)	13 (27.7)	14 (9.6)	7 (30.4)	0.04	0.81	0.46	< 0.01

Data are presented as the number or mean ± S.D. HbA1c: hemoglobin A1c. HDL: high-density lipoprotein. *P*_1_: comparisons of respective variables between genders in nonsmokers, *P*_2_: comparisons of respective variables between genders in current smokers, *P*_3_: comparisons of respective variables between nonsmokers and current smokers in males, *P*_4_: comparisons of respective variables between nonsmokers and current smokers in females (using unpaired *t*-tests and Chi-square tests).

The results of the unadjusted, age-adjusted, and multivariate-adjusted logistic regression analyses for OA are shown in Table 2. In males, systolic blood pressure was independently, significantly, and positively associated with OA. Diastolic blood pressure was significantly and positively associated with OA in the unadjusted and age-adjusted analyses, but it was no longer significantly associated with OA in the multivariate-adjusted logistic regression analysis. The other variables, including current smoking status, were not significantly associated with OA. In females, systolic blood pressure was also independently, significantly, and positively associated with OA. Current smoking status was found to be significantly and positively associated with OA in the unadjusted and age-adjusted analyses. The multivariate-adjusted logistic regression analysis revealed that current smoking status was independently, significantly, and positively associated with OA (Odds Ratio, 5.41; 95% Confidence Interval, 1.69–17.30; *P* < 0.01).

**Table 2:** Logistic regression analysis for urinary protein (≥ 300 mg/g creatinine)

	**Male (n = 90)**						**Female (n = 169)**					
	**Unadjusted**		**Age-adjusted**		**Multi-adjusted**		**Unadjusted**		**Age-adjusted**		**Multi-adjusted**	
	**OR (95% CI)**	***P***	**OR (95% CI)**	***P***	**OR (95% CI)**	***P***	**OR (95% CI)**	***P***	**OR (95% CI)**	***P***	**OR (95% CI)**	***P***
Age (years)	1.00 (0.97–1.04)	0.81	-		1.00 (0.96–1.04)	0.85	0.99 (0.97–1.02)	0.48	-		0.96 (0.92–1.00)	0.07
BMI (kg/m^2^)	1.10 (0.94–1.28)	0.23	1.10 (0.94–1.28)	0.22	1.04 (0.84–1.23)	0.72	0.97 (0.85–1.10)	0.59	0.97 (0.86–1.10)	0.62	0.96 (0.83–1.10)	0.52
SBP (mmHg)	1.03 (1.01–1.06)	< 0.01	1.03 (1.01–1.06)	< 0.01	1.04 (1.00–1.08)	0.03	1.03 (1.01–1.06)	0.02	1.05 (1.01–1.08)	< 0.01	1.06 (1.02–1.11)	0.01
DBP (mmHg)	1.04 (1.00–1.08)	0.04	1.04 (1.00–1.08)	0.04	0.99 (0.94–1.05)	0.78	1.03 (0.99–1.07)	0.18	1.03 (0.99–1.07)	0.21	0.98 (0.93–1.03)	0.44
HbA1c (%)	0.99 (0.76–1.29)	0.95	0.99 (0.77–1.29)	0.96	1.05 (0.76–1.46)	0.78	1.03 (0.83–1.27)	0.81	1.01 (0.82–1.26)	0.90	1.04 (0.77–1.39)	0.81
TC (mg/dL)	1.01 (0.99–1.03)	0.09	1.01 (0.99–1.03)	0.09	1.01 (0.99–1.03)	0.21	1.00 (0.98–1.01)	0.57	0.99 (0.98–1.01)	0.54	0.99 (0.97–1.01)	0.26
Triglyceride (mg/dL)	1.00 (0.99–1.01)	0.42	1.00 (0.99–1.01)	0.39	1.00 (0.99–1.01)	0.61	1.00 (0.99–1.01)	0.84	1.00 (0.99–1.01)	0.76	1.00 (0.99–1.01)	0.87
HDL-C (mg/dL)	1.01 (0.99–1.03)	0.46	1.01 (0.99–1.03)	0.48	0.99 (0.95–1.02)	0.44	1.00 (0.97–1.02)	0.80	1.00 (0.97–1.02)	0.80	1.02 (0.98–1.06)	0.37
Current smoking	1.44 (0.55–3.83)	0.46	1.48 (0.55–3.97)	0.44	1.15 (0.37–3.50)	0.81	4.13 (1.45–11.73)	< 0.01	4.00 (1.40–11.47)	0.01	5.41 (1.69–17.30)	< 0.01

OR: odds ratio. CI: confidence interval. BMI: body mass index. SBP: systolic blood pressure. DBP: diastolic blood pressure. HbA1c: hemoglobin A1c. TC: Total cholesterol. HDL-C: high-density lipoprotein-cholesterol. Multivariate-adjusted analysis models include age, BMI, SBP, DBP, HbA1c, TC, triglyceride, HDL-C, and current smoking status.

## Discussion

In the present study, the female T1DM group had a lower prevalence of OA than the male T1DM group. Furthermore, current smoking status was significantly associated with urinary OA in female T1DM patients, while no factor was significantly associated with OA in male T1DM patients. It is well known that the avoidance of smoking has beneficial health effects in patients with diabetes [13,14]. Furthermore, the proportion of smokers in our country has been gradually decreasing as the prevalence of smoking in patients with diabetes was high, especially in young female T1DM patients [15]. Based on these results, we propose that female patients with T1DM should cease smoking in order to prevent DN.

Smoking increases the concentration of carboxyhemoglobin, platelet aggregability, and the fibrinogen concentration, all of which cause tissue hypoxia and contribute to vascular damage [16,17], especially that caused by diabetes [18]. Glomerular hyperfiltration [19], the suppression of water excretion [20], and the effects of proximal tubular damage [21] from smoking are the mediators of DN progression.

In this study, we found a relatively stronger correlation between smoking and DN in females than in males. However, the underlying mechanisms for this remain unclear. Smoking can decrease the atheroprotective effects of estrogens [22,23]. Smoking appears to alter estradiol metabolism, leading to the increased production of inactive catechols [23]. This may be related to the induction of estrogen-metabolizing cytochrome P450 isoenzymes [24]; furthermore, smoking lowers estrogen activity [25,26]. The effects of estrogen dysfunction on DN progression might be greater than the effects of a lowered amount of estrogen (probably corresponding to male patients). The oxidative milieu can result in proinflammatory effects of HDL [27,28]. In this study, HDL-C levels in female patients were high relative to male patients (particularly in nonsmokers); however, instead of affecting the actual HDL-C levels, diabetic states might make HDL dysfunctional. Functional studies to verify these hypotheses are warranted.

Females were more likely to be assigned to a lower-risk category than males, even though the former are at an equivalent calculated risk [8]. Probably, a higher proportion of females compared to males develop DN from T1DM because of a lack of awareness and a systematic delay in the diagnosis of complications. In Japan, a small percentage of female individuals exhibit smoking habits [15]; hence, this situation is less alarming for female patients compared to male patients.

This study had a limitation related to the interpretation of data that warrants mention. First, the sample size of the groups may have been too small to produce definite conclusions. Moreover, it is necessary to consider that this study was a cross-sectional study and was only conducted at a single center. This study was performed in a local university hospital, which might not be representative of the entire T1DM population in Japan. One possibility is that T1DM patients who had undergone kidney dialysis, especially males, would discontinue visiting our hospital. Moreover, patients would visit our hospital when the complications associated with diabetes had become severe and progressive, as mentioned in a previous report [29]. Therefore, a multicenter study with a large number of patients is required. Second, because the dose of smoking [5,30], the duration of smoking, and the type of cigarette [31] were significantly associated with the progression of nephropathy, we might have to investigate these factors in detail. In addition, the self-reporting of smoking habits can be questionable because of underreporting.

## Conclusions

In conclusion, the present study suggested that current smoking status can promote renal dysfunction in female T1DM patients, and we may need to take into account an individual’s smoking status in the management of T1DM, especially in females.

## Abbreviations

T1DM: Type 1 diabetes mellitus; DN: Diabetic nephropathy; NA: Normoalbuminuria; MA: Microalbuminuria; OA: Overt albuminuria; ACR: Albumin/creatinine ratio; Cr: Creatinine; HbA1c: Hemoglobin A1c; BMI: Body mass index; TC: Total cholesterol; TG: Triglyceride; HDL-C: High-density lipoprotein cholesterol; SD: Standard deviation.

## Competing interests

The authors declare that they have no competing interests.

## Authors’ contributions

KO researched the data, wrote the manuscript and contributed to the discussion; KK analyzed the data and edited and reviewed the manuscript; MM provided the data; HY, JO, and SN contributed to the discussion; and SI contributed to the discussion and reviewed the manuscript. All authors read and approved the final manuscript.
